# Cell Lysis and Detoxification of Cyanotoxins Using a Novel Combination of Microbubble Generation and Plasma Microreactor Technology for Ozonation

**DOI:** 10.3389/fmicb.2018.00678

**Published:** 2018-04-05

**Authors:** Jagroop Pandhal, Anggun Siswanto, Dmitriy Kuvshinov, William B. Zimmerman, Linda Lawton, Christine Edwards

**Affiliations:** ^1^Department of Chemical and Biological Engineering, University of Sheffield, Sheffield, United Kingdom; ^2^Vocational School, Diponegoro University, Semarang, Indonesia; ^3^School of Engineering and Computer Science, University of Hull, Kingston Upon Hull, United Kingdom; ^4^School of Pharmacy and Life Sciences, Robert Gordon University, Aberdeen, United Kingdom

**Keywords:** harmful algal blooms, cyanobacteria, cyanotoxins, microbubbles, ozonolysis, plasma microreactor

## Abstract

There has been a steady rise in the incidences of algal blooms globally, and worryingly, there is increasing evidence that changes in the global climate are leading to a shift toward cyanobacterial blooms. Many cyanobacterial genera are harmful, producing several potent toxins, including microcystins, for which there are over 90 described analogues. There are a wide range of negative effects associated with these toxins including gastroenteritis, cytotoxicity, hepatotoxicity and neurotoxicity. Although a variety of oxidation based treatment methods have been described, ozonation and advanced oxidation are acknowledged as most effective as they readily oxidise microcystins to non-toxic degradation products. However, most ozonation technologies have challenges for scale up including high costs and sub-optimum efficiencies, hence, a low cost and scalable ozonation technology is needed. Here we designed a low temperature plasma dielectric barrier discharge (DBD) reactor with an incorporated fluidic oscillator for microbubble delivery of ozone. Both technologies have the potential to drastically reduce the costs of ozonation at scale. Mass spectrometry analysis revealed very rapid (<2 min) destruction of two pure microcystins (MC-LR and MC-RR), together with removal of by-products even at low flow rate 1 L min^−1^ where bubble size was 0.56–0.6 mm and the ozone concentration within the liquid was 20 ppm. Toxicity levels were calculated through protein phosphatase inhibition assays and indicated loss of toxicity as well as confirming the by-products were also non-toxic. Finally, treatment of whole *Microcystis aeruginosa* cells showed that even at these very low ozone levels, cells can be killed and toxins (MC-LR and Desmethyl MC-LR) removed. Little change was observed in the first 20 min of treatment followed by rapid increase in extracellular toxins, indicating cell lysis, with most significant release at the higher 3 L min^−1^ flow rate compared to 1 L min^−1^. This lab-scale investigation demonstrates the potential of the novel plasma micro reactor with applications for *in situ* treatment of harmful algal blooms and cyanotoxins.

## Introduction

Although the specific interacting parameters and mechanisms that have led to an increase in incidences of harmful algal blooms (HABs) remain diverse and not fully understood, the impact of human activity through global warming and rapid industrialisation is becoming more apparent. Globally, the number of lakes with HABs is predicted to increase by at least 20% until 2050 (UNDESA, 2012). HABs result largely from nutrient enrichment, but also from other physicochemical changes, leading to overgrowth of cyanobacteria cells capable of producing toxins (i.e., cyanotoxins). These toxins present a hazard to animal and human health. In the developing world, many of the affected freshwater ponds are essential for bathing, clothes washing, recreation, cattle drinking and irrigation, enhancing the opportunities for direct exposure. Direct exposure may result from oral ingestion of cyanotoxins via contaminated water or inhalation during recreation/other direct water use. Indirect exposure via food is also a potential route for chronic exposure (van Apeldoorn et al., 2007). Although reduction of nutrient enrichment is a primary objective to reduce HAB events, it is not always possible due to diffuse pollution or rapidly fluctuating water levels. *In situ* treatments are particularly difficult to implement, and any rising occurrences of HABs will be particularly catastrophic for countries where access to clean drinking water is already a considerable challenge.

Cyanotoxins, for example, microcystins (MCs), are cyclic peptides often associated with blooms of cyanobacterial genera including *Microcystis, Anabaena*, and *Oscillatoria* (Carmichael, 1992). They are relatively stable when released into the water and have been linked to acute and chronic toxicities (van Apeldoorn et al., 2007). MCs are transported to liver cells where they can inhibit protein phosphatases, leading to haemorrhagic shock. As hepatoxins they can promote tumour growth and neurological effects (Svrcek and Smith, 2004). Although there are over 200 variants, MCs have the general structure of three standard amino acids, alanine (Ala), methylaspartic acid (MeAsp) and glutamic acid (Glu), including two unusual amino acids *N*-methyldehydroalanine (Mdha) and 3-amino-9-methoxy-2,6,8-trimethyl-10-phenyldeca-4,6-dienoicacid (Adda), with the latter being responsible for the biological activity of the toxins (Rinehart et al., 1988).

A variety of methods have been tested to both break down dissolved cyanotoxins within water and also to lyse cyanobacterial cells to enable detoxification of released toxins. Oxidation through addition of potassium permanganate has been shown to remove MCs, although not necessarily lyse cyanobacterial cells (Lam et al., 1995). The dose and contact time are significant variables with its application, as it is with addition of chlorine. More problematic with chlorine applications is the generation of toxic by-products, e.g., chloroform (Tibbetts, 1995). Physical methods such as activated carbon adsorption, membrane filtration and biofilm treatment through sand filters, have been demonstrated to successfully decontaminate water, however, problems still persist due to incomplete degradation of toxins, high costs or slow removal rates (Falconer et al., 1989; Mesquita et al., 2006; Walker, 2014). Issues surrounding current methods are the cost of application, use of unsustainably sourced chemicals and potential negative impacts on the ecosystem. An environmentally friendly and green solution to reduce levels of cyanotoxins below World Health Organisation recommended limits (<1 μgL^−1^ MC in drinking water) is urgently required (WHO, 1998).

These significant drawbacks as well as the prediction of more prevalent toxic blooms has led researchers to look for alternative cyanotoxins degradation technologies, with advanced oxidation processes (AOPs) receiving increasing attention. AOPs have been tested for the degradation of a wide variety of organic compounds, with application in wastewater treatment, aquaculture, food processing and effluent treatment (Suty et al., 2004; Vogelpohl and Kim, 2004). The process requires the generation of highly oxidising hydroxyl radicals (•OH) through application of primary oxidants (e.g., ozone, O_3_ and hydrogen peroxide, H_2_O_2_), energy (e.g., UV light) and catalysts (e.g., titanium dioxide, TiO_2_), which attack specific bonds leading to rapid degradation of toxin molecules. Environmental concerns of chemical addition have been somewhat overcome by selected AOP's, particularly ozonation, which can be efficient at high concentrations of cyanobacterial cells, for cell lysis and degradation of cyanotoxins. It is recognised as a clean technology as unreacted ozone molecules will dissociate back into oxygen. Ozone attacks the double bonds and amine moieties in MCs. The impact of ozone on cyanotoxin concentrations in drinking water has been classified as fast for MiCs, anatoxin-a and cylindrospermopsin (Fawell et al., 1993; Rositano et al., 2001; Newcombe and Nicholson, 2004; Onstad et al., 2007; Cheng et al., 2009) at 20°C and pH 7, with fast achieving 90% oxidation in less than 10 min. Onstad et al. determined the second order rate constants for the reaction of cyanotoxins with ozone and H_2_O_2_ to determine optimal conditions for decontamination of natural waters, and highlighted how ozone primarily attacks the structural features of MC-LR that are responsible for its toxic effects (Onstad et al., 2007). Hence, ozone is considered a very effective treatment to remove cyanotoxins.

One of the drawbacks of using ozone for cyanotoxins degradation is the associated cost of the application, which includes capital investment, maintenance and energy requirements. Dore et al. (2013) estimated the average costs of a variety of oxidation methods and found it to have the highest cost based on water treatment plants with a capacity of treating 100–5,000 m^3^ d^−1^, followed by being the second most expensive to UV treatment when used at 10,000–50,000 m^3^ d^−1^ (Dore et al., 2013). Despite implying that ozone treatment is a feasible treatment option at large-scale, a reduction in overall function costs would be a desirable development.

Here we combine ozone generation with the use of a bespoke low temperature plasma dielectric barrier discharge (DBD) reactor (Kuvshinov et al., 2014) and fluidic oscillator microbubble technology (Zimmerman et al., 2011). Construction of this DBD reactor and the diffuser permits fast delivery of the formed ozone to the reaction volume in form of microbubbles. Bubbles are classified as microbubbles when in the size range of 1–999 μm, and this high surface area to volume ratio provides fast heat and mass transfer, as well as longer residence time (Muroyama et al., 2013). Due to microbubbles staying longer in the bulk of liquid, increasing the contact time between the gas and the liquid, they could potentially increase the rate of cyanotoxins degradation. Standard microbubble generation is energy intensive and therefore we integrated fluidic oscillator technology, a synthetic hybrid jet microfluidic device with no moving parts (Zimmerman et al., 2011).

In this work, our novel cyanotoxin detoxification technology was characterised at laboratory scale, measuring the degradation rate of two key MCs, MC-LR, and MC-RR. Mass spectrometry and a protein phosphatase assay were used to investigate toxicity levels and bi product production, followed by an examination of the impact on intact *Microcystis aeroginosa* cells.

## Materials and methods

### Plasma microreactor design

**Figure 1:**
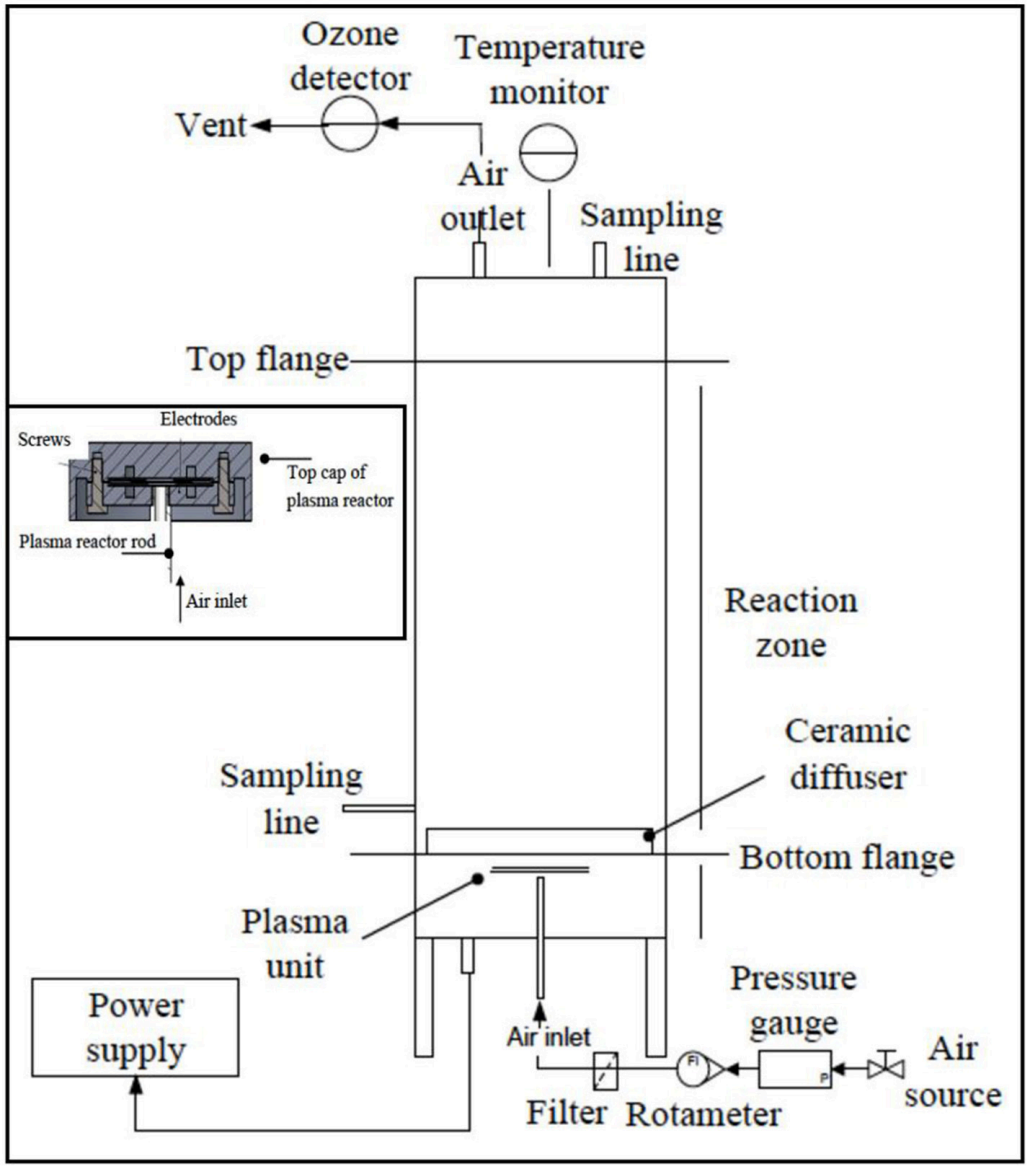
The experimental plasma microreactor. The suggested working voltage level was 4 kV with the plasma unit connected to a 500 W/50 kHz/4 kV plasma power supply (Dipl.Ing.H.Bayerle, Germany) Inset: A diagram of the experimental reactor performing ozonolysis.

An overview of the plasma reactor design is presented on Figure 1. The bespoke setup consisted of an experimental reactor, a power supply and flow network. The 2 L reactor has a stainless steel cylindrical body with a plasma unit, with connectors on the top for an air exhaust, a temperature monitor and a sampling line. The plasma unit, a standalone device, is attached to the base of the reactor, providing an inlet. The plasma unit comprises the round shape DBD plasma microreactor and a ceramic diffuser. The air enters the centre of the plasma microreactor via a hollow supporting rod flows radially into a 300 μm gap formed by two coaxial parallel circular copper electrodes, 22 mm in diameter, covered by 140 μm thick glass wafers (Thermo Scientific, Menzo Glässer). The inter-electrode gap and the footprint of the electrodes limit the plasma volume. Ozone is generated from air within the specified DBD conditions. At the gap exit, the flow of the ozone-air mixture goes up through the ceramic porous plate of the diffuser and enters the reaction volume in form of bubbles. The disc shape ceramic plate with diameter of 7.2 cm and thickness of 0.5 cm, is made of sintered porous alumina and silica (80:20 w/w) with 20 μm pore size (HP Technical Ceramic, Sheffield, United Kingdom). The gas in-flow was controlled by a rotameter and a pressure gauge. Ozone presence was qualitatively monitored using the ozone detector (EcoZone Monitor, Model EZ-1X; Eco Sensor, USA) incorporated to the gas(air) outlet line at the top of the reactor. The plasma microreactor was powered by a 500 W, 50 kHz, 4 kV plasma power supply (Dipl.Ing.H.Bayerle, Germany) and equipped with a second transformer Trafo FE 48 V, 4 kV. The power level was monitored with a combination of a PC controlled oscilloscope Picoscope ADC-212 and the high voltage probe TES TEC HVP-15 HF. The obtained signal was further processed with the Picoscope 5.21.1 software. Here, the reactor was used in batch mode, although it can be adapted for continuous treatment.

### Ozone concentration and bubble size measurement

The ozone concentrations in liquid and gas phases were measured by the Indigo method (Bader and Hoigné, 1981). The absorbance of the decolourised indigo was measured at wavelength of 600 nm with the use of DR 2800 spectrophotometer (Hach-Lange, Colorado USA). A calibration curve was performed prior to experimentation (Supplementary Material Figure S4). Equation (1) presents the calculation of indigo concentration in the stock solution:

(1) $$\begin{array}{r} {\text{C}}_{\text{ss}}=\frac{{\text{m}}_{\text{pi}}}{\text{m}{\text{w}}_{\text{pi}}}\times \frac{1}{{\text{V}}_{\text{T}}} \end{array}$$

where Css is the concentration of stock solution (mol L^−1^), mpi is the mass of potassium indigo (g), mwpi is the molecular weight of potassium indigo (g mol^−1^) and VT is the total volume of the mixture (L).

Measurement of ozone content in the gas phase was conducted by sampling from the gas(air) outlet line. Sampling was performed by flushing the outgoing gas ozone containing the mixture through a washing flask filled with indigo solution for 30 s. The change in absorbance of the indigo solution before and after bubbling provided the concentration of ozone in the gas phase. For ozone concentration measurement in the liquid phase, an in-house technique was applied. Briefly, a 4 mL sample of ozone containing liquid was taken from the side sampling line of the reactor using a syringe preloaded with 4 mL of Indigo solution. The mixture was homogenised by vibrating for 30 s, and was subsequently analysed by spectrophotometry. As previously, the change in absorbance of the Indigo solution before and after sampling gave the concentration of the ozone in the liquid phase, but this time taking the dilution factor (1:1) into account.

The bubble formation process for the applied ceramic diffuser of the plasma unit was characterised by high-speed photography, where the micro plasma reactor was placed in a transparent glass tank filled with water. The airflow to the unit varied in the range of 0.5–3 L min^−1^ and was operated by a 0–5 L min^−1^ flow controller (Key Instruments, Cole-Parmer, Illinois USA). To film the process the FastCam HS3 Photron camera (Chicago, USA) equipped with Nikon AF Lens was used. The camera was computer controlled by Photron Fastcam Viewer (PFV) Software (Version 2410). To calculate bubble size distribution, open source software ImageJ, was used. Bubble size distribution data was analysed statistically by varying the diameter of the microbubbles and plotting this as a function of corresponding population. Although bubble size distributions is often characterised by their mean and standard deviation, standard deviation often only represents the variability of bubble average size but not the range of bubble size distribution. Here, span value was used (Equation 2), a dimensionless unit where smaller value represents narrower range of distribution:

(2) $$\begin{array}{r} Span=\frac{D_{b}^{90}-D_{b}^{10}}{D_{b}^{50}} \end{array}$$

where $D_{b}^{90}$ corresponds to the bubble diameter of the 90th centile, $D_{b}^{10}$ is defined as the 10th centile, while $D_{b}^{50}$ is the median bubble diameter. These values are obtained from the cumulative bubble population distribution.

### Cyanobacterial cells

*Microcystis aeruginosa* PCC 7806 (Pasteur Culture Collection, Paris, France) was grown on BG11 medium in 10 L glass vessels at a temperature of range of 22°C ± 2 with continuous light at ~20 μmol photons m^−2^ s^−1^) for 4 weeks.

### Ozone application

The impact of ozone was investigated on two commonly found dissolved microcystins (MC-LR and MC-RR) and on whole *M. aeruginosa* PCC 7806 cells, which prominently contain MC-LR and DM-LR microcystins. Dissolved microcystins were obtained from Enzo Life Sciences (Exeter, UK). MC-LR was dissolved in 0.5 L distilled water to give a final concentration of 2 mg L^−1^, placed in the plasma microreactor and treated with ozone microbubbles at a flow rate of 1 L min^−1^. Samples were taken prior to application of microbubbles and at 2 min intervals for 20 min and stored at 4°C until analysis by ultra-performance liquid chromatography-mass spectrometry (UPLC-MS). The process was repeated using airflow rates of 2 and 3 L min^−1^. The described protocol was then applied to MC-RR at the same concentration of 2 mg L^−1^.

A coulter counter (Multi-sizer3, Beckman Life Science, Hertfordshire, UK) was used to measure cell density. Briefly, the sample was diluted with isotone (0.1% v/v) prior to analysis and a blank (BG11 media) run through the coulter counter. The samples were then analysed and the blank subtracted. The cells per ml, within the ranges of 2–4 μm, were recorded. For experiments using whole cells, 0.5 L of *M. aeruginosa* at a density of ~12 million cells ml^−1^ were placed in the reactor and treated with ozone microbubbles at a flow rate of 1 L, 2 L, and 3 L min^−1^, with sampling every 2 min for 20 min, then at 30 and 60 min. Samples were centrifuged at 13,000 × g, supernatants were removed and stored separately for analysis of intracellular and extracellular MC. Pellets were extracted in 1 ml aqueous methanol (75% v/v) for 1 h and further centrifuged at 13,000 × g for 10 min. This supernatant was removed for intracellular MC analysis.

Triplicate runs enabled a standard deviation calculation, and to obtain statistical information on the varied flow rates (*p*-≥ 0.05), an unpaired *t*-test with Welch's correction, assuming both the populations do not have the same standard deviation, was run using the analysis tool on GraphPad. Only statistical significant differences were discussed in section Results and Discussion.

### UPLC analysis of microcystins

An ACQUITY UPLC system with an ACQUITY photodiode array detector (PDA) coupled with a Xevo Quadrupole time of flight (QTOF) mass spectrometer in series (Waters, Elstree, UK) fitted with an ACQUITY UPLC CORTECS C18 column (2.1 mm diameter, 100 mm length, 1.7 mm particle size) (Waters, Elstree, UK) was used to measure MC concentrations. During PDA detection, samples were monitored from 200 to 400 nm with a resolution of 1.2 nm. For mass spectrometry analysis, positive electro-spray ionisation (ESI+) was used, the detector scanned from m/z 50 to m/z 2000 Da with a scan time of 0.25 s and an inter-scanner delay time of 0.025 s. The capillary voltage applied was 3.0 kV and cone voltage was set to 25 V. The source and desolvation temperatures were 80 and 300°C respectively. The flow rate for the cone gas was set to 50 L h^−1^ and the flow rate of the desolvation gas was set to 40 L h^−1^. For the buffers, high purity water (Elga, High Wycombe, UK) and acetonitrile containing 0.1% formic acid was used. Separation was achieved using a gradient starting with 20% acetonitrile, which increased to 80% over 10 min, followed by a washing step (100% acetonitrile) and re-equilibration over the next 5 min. Total gradient time was 15 min with a flow-rate of 0.2 mL min^−1^. Microcystins were quantified by extraction at 238 nm and metabolites identified by mass spectrometry.

### Determination of toxicity

Inhibition of protein phosphatase 1 was determined using paranitrophenol (pNPP) as the substrate (Sigma, Poole, UK), as described previously (Ward et al., 1997). Briefly, buffers were prepared to give protein phosphatase 1 and pNpp at working concentrations of 5.0 μg mL^−1^ and 5 mM respectively. Prior to evaluation of ozone treated samples, calibration curves for both MC-RR and MC-LR were generated in the range of 3 ng mL^−1^ to 1 μg mL^−1^. Standards and samples were incubated with the substrate along with appropriate controls and blanks in 96 well mictotitre plates at 37°C for 1 h. Plates were read at 405 nm using an Epoc plate reader (Biotek, UK) with Gen5 version 2.04.11 software. Percentage inhibition was calculated from the readings.

## Results and discussion

### Ozone concentration and bubble size distribution

The plasma microreactor performance was characterised by ozone concentration measurement using the Indigo method (Bader and Hoigné, 1981). This method is widely accepted as a sensitive, precise and fast technique to determine the ozone concentration. The indigo solution in the samples was discoloured indicating that bonds were cleaved by the presence and action of ozone. The calculation of ozone concentration using the indigo reagent is based on a ratio where 1 mol of decolourised indigo is equal to 1 mol of ozone detected, assuming that the applied potassium indigo trisulfonate was pure. The experimental data on the ozone concentration from the plasma microreactor at the constant applied voltage of 4.4 kV and the flow rate ranges (1, 2, and 3 L min^−1^) showed zero ozone concentration (below the control method detection limit) in the gas (air) output line. The ozone concentration in the liquid phase was detected at the level of 20 ppm at 1 L min^−1^, 27 ppm at 2 L min^−1^ and 26 ppm at 3 L min^−1^ (Supplementary Material Figure S1).

Microbubbles are an attractive option for a wide variety of industries, including aeration, separation and de-emulsification. Although high-energy intensive microbubble generation is used within wastewater treatment, there is increasing demand to develop technologies to reduce energy requirement to extend the use of microbubbles to industries where gas-liquid processes are used. The generation of microbubbles using fluidic oscillation with appropriate diffusers has shown a significant improvement in bubble throughput, including a reduction in bubble size, without the concomitant expenditure of high energy (Zimmerman et al., 2011). This process is therefore a highly sustainable and economic way to generate microbubbles, particularly for large-scale applications, including treatment of cyanotoxins containing lakes or water retention ponds. Microbubbles must be smaller than 1 mm and larger than 1 μm size in diameter and therefore size measurements were undertaken. It is also important to evaluate how airflow rate relates to bubble size. Although several techniques can be used to characterise bubble size, including photonic, acoustic and optical (Vagle and Farmer, 1998), in this study, size distribution was measured from multiple images taken using a FastCam HS3 Photron camera, with a combination of PFV and ImageJ software. The bubble size distribution is shown in Figure S2 (Supplementary Materials) and shows a Gaussian distribution shape, with the smaller size range corresponding to the lower flow rate. Bubble sizes were 0.56–0.60 mm for 1 L min^−1^, 0.66–0.70 mm for 2 L min^−1^ and 0.76–0.80 mm for 3 L min^−1^. Therefore, all three flow rates were able to produce microbubbles.

Although increasing the flow rate from 1 L min^−1^ to 2 or 3 L min^−1^ increases ozone concentration by ~30–35%, the energy consumption of doubling or tripling flow rates might not be offset by the impacts of such a small change in ozone concentrations. To evaluate this impact of the different flow rates, treatment of dissolved MC standards and whole *M. auruginosa* cells were undertaken.

### Ozone treatment of dissolved microcystins

**Figure 2:**
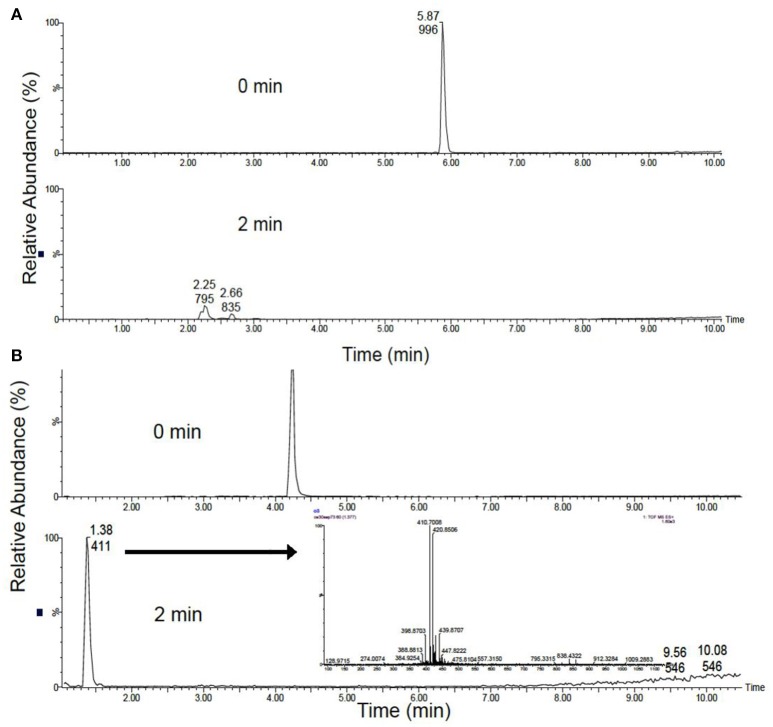
Scaled total ion chromatograms at a flow rate of 1 L min^−1^, equivalent to 0.199 ppm O_3._ **(A)** MC-LR before (top) and after 2 m (bottom) of treatment **(B)** MC-RR before (top) and after 2 m (bottom) of treatment. Inset: The spectra of unknown intermediates, unseparated peaks were detected in the void volume, m/z 411 and 421.

**Figure 3:**
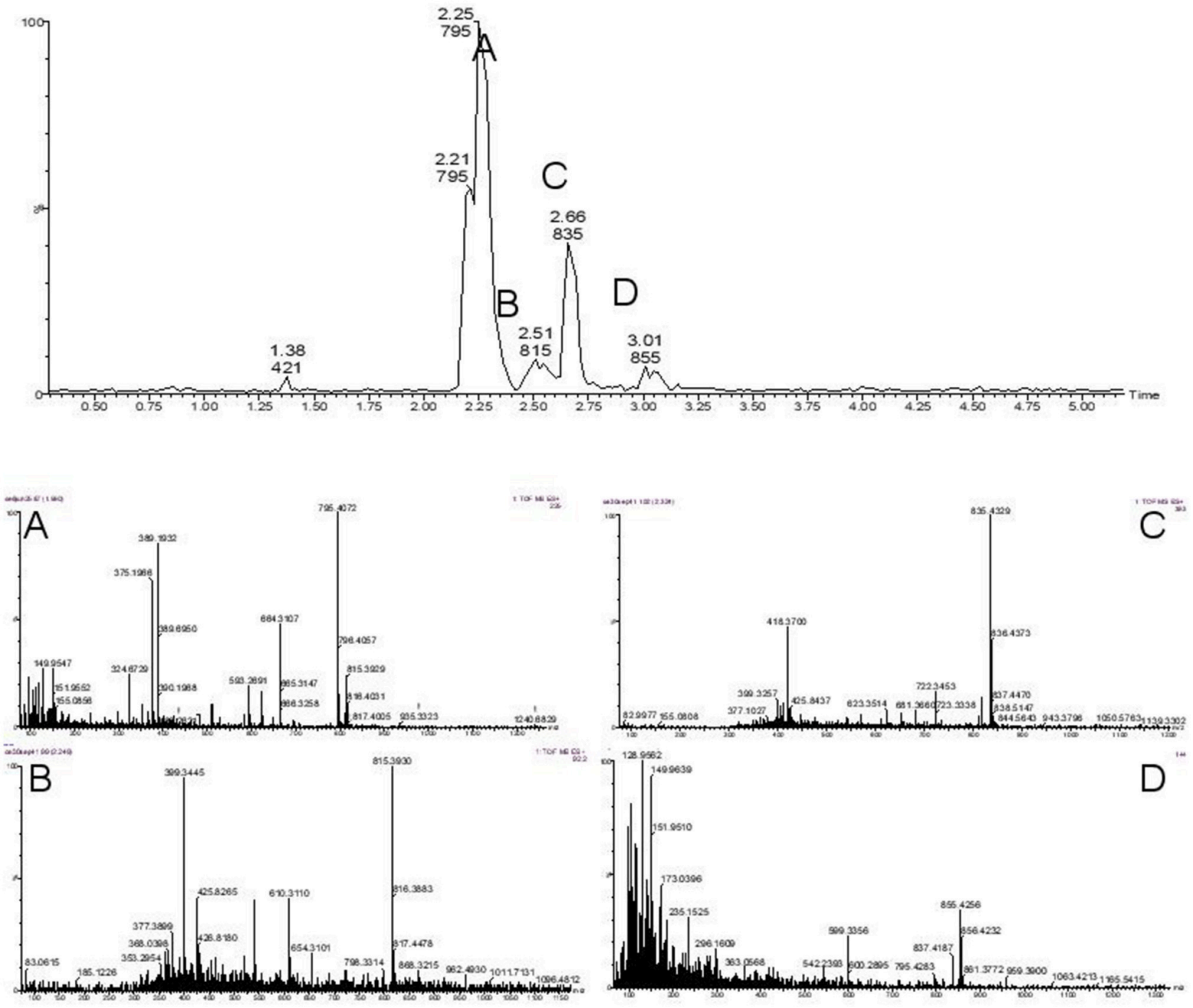
Identification of four commonly observed degradation intermediates of MC-LR during treatment with ozone microbubbles. The expanded mass chromatogram shows four clear peaks A with isomers m/z 795, B m/z 815, C m/z 835 and D m/z 855.

**Figure 4:**
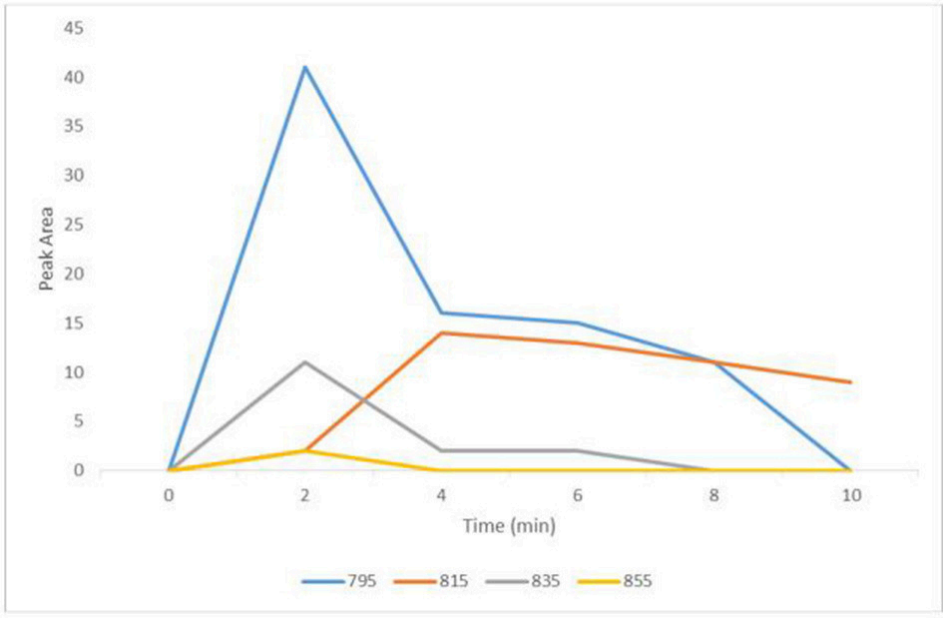
Degradation intermediates of MC-LR during microbubble treatment at a flow rate of 1 L min^−1^ equivalent to 0.199 ppm O_3_. Peak A, 795 (blue); Peak B, 815 (orange); Peak C, 835 (gray); Peak D, 855 (yellow).

Complete degradation of MCs was observed in all conditions demonstrating the strong oxidizing capability of the system. MC-LR was completely destroyed at the lowest 1 L min^−1^ flow rate after only 2 min (Figure 2A), with the formation of common degradation intermediates. Figure 3 shows the identification of the four commonly observed degradation intermediates of MC-LR in an expanded mass chromatogram with multiple peaks at *m/z* 795, typically attributed to geometric isomers, and the key intermediates at *m/z* 815, 835, and 855, resulting from initial dihydroxylation of the diene bonds (C_4_-C_5_ and C_6_-C_7_) of the Adda amino acid, followed by further oxidative cleavage steps (Chang et al., 2014). No peak at *m/z* 1030, representing Cyclo [(OH_2_)Adda-Glu-Mdha_Ala-Leu-MeAsp-Arg-H], the primary product of MC-LR ozonation was detected, this was most likely due to the relatively low concentrations of test MCs. Very similar results were obtained for the higher flow rate (2 and 3 L min^−1^) samples. Although unseparated peaks were detected in the void volume, *m/z* 411 and 421 were not identified. Interestingly, the area of these peaks only reduced by 25% over 10 min at 1 L min^−1^, and treatments with higher flow rates resulted in 50% reduction in peak area after 10 min but showed insignificant change from this after 20 min. Figure 4 shows the change in peak area intensity of the four intermediates over time. Peak A, m/z 795, consistent with the Cyclo [C_7_H_7_O_2_N-Glu-Mdha_Ala-Leu-MeAsp-Arg-H] from oxidation of C_4_-C_5_ bond in Adda, was the most abundant intermediate after 2 min but gradually declined and was undetectable by 10 min of treatment. Peak B, *m/z* 815, a further oxidation product of *m/z* 795, most likely at site of Mdha, increased during treatment and was still present at 10 min, and therefore relatively stable to treatment. Peak C, *m/z* 835, most likely a result of C_6_-C_7_, giving cyclic peptide intermediate Cyclo [C_8_H_11_O_2_N-Glu-Mdha_Ala-Leu-MeAsp-Arg-H], declined from 2 min and was undetectable by 8 min and finally, Peak D (*m/z* 855), attributed to oxidation of *m/z* 835 at Mdha, was only detected in 2 min samples.

MC-RR was also rapidly destroyed, but unlike with MC-LR, characteristic intermediates were not detected, highlighting the different behaviour of microcystin variants (Figure 2B). Since primary attack of oxidation is the unconjugated double bond on Adda, the rapid destruction of both MCs was consistent with published work (Miao et al., 2010). In contrast to this work MC-RR was more rapidly degraded than MC-LR with no detection of characteristic by-products. In addition, MC concentration in the study by Maio et al. was 50 mg L^−1^ compared to the 2 mg L^−1^ used for the current study, which would undoubtedly impact oxidation kinetics, the potential formation and detection of by-products and elucidation of the degradation pathways.

### Phosphatase inhibition assay

In order to evaluate the inhibitory activity of the MC by-products generated during the ozonation process, assays based on protein phosphatase 1 (Sigma, Poole, UK) were undertaken over the treatment period. The IC_50_ of MC-LR and MC-RR were both determined as ~10 ng ml^−1^, in the range consistent with published values (Ward et al., 1997; Rapala et al., 2002).

**Figure 5:**
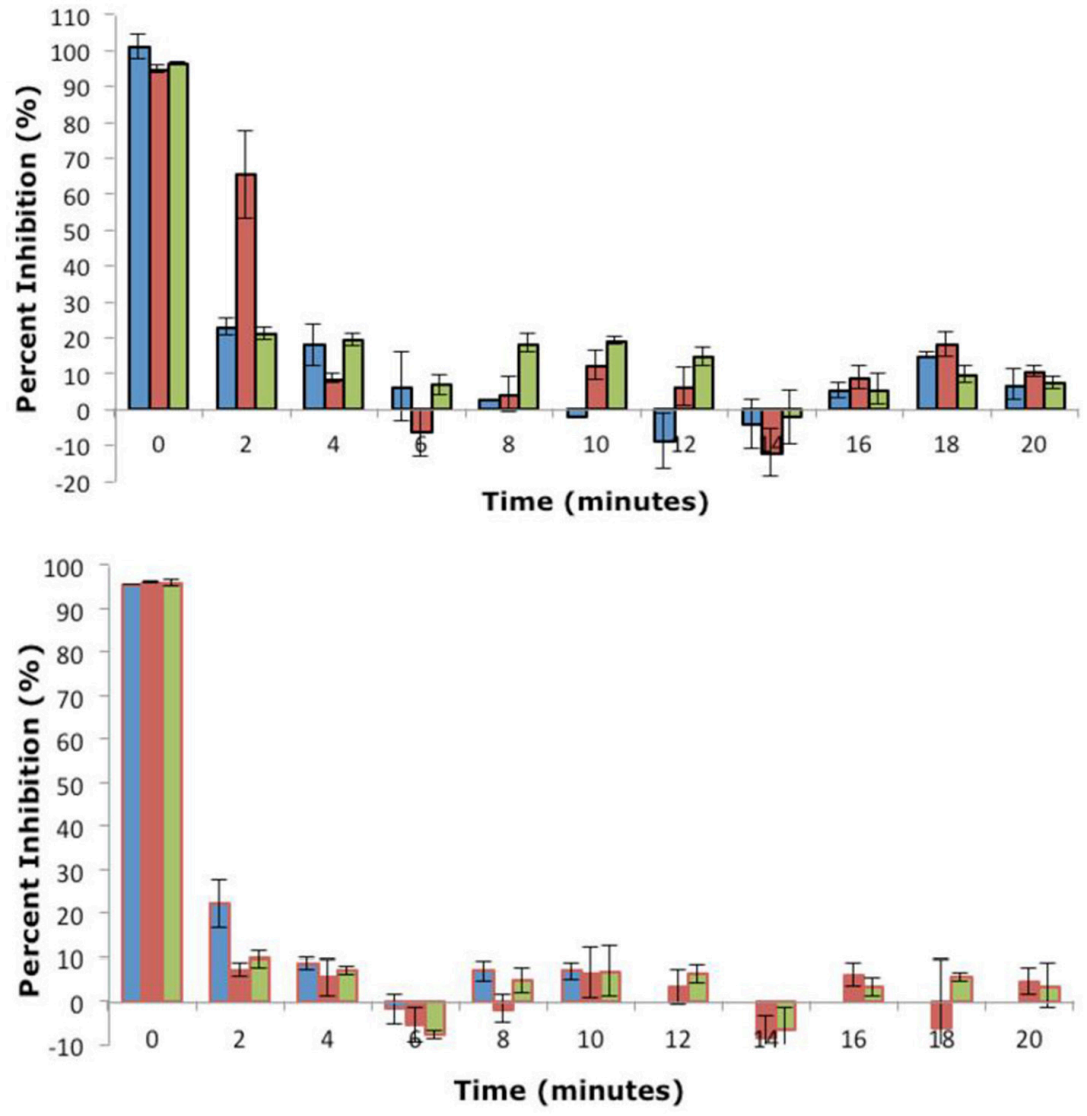
Inhibition of MC ozonolysis products against protein Phosphatase 1 after treatment at an air flow 1 L min^−1^ (0.199 ppm O_3_; 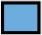), 2 L min^−1^ (0.265 ppm O_3_; 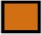) and 3 L min^−1^ (0.258 ppm O_3_; 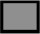). Microtitre plates were read at 405 nm after incubation at 37°C for 1 h. Data plotted is mean of three readings and vertical bars represent standard deviation. Top: MC-LR, Bottom: LC-RR (*n* = 3).

The rapid removal of both MC-LR and MC-RR observed by UPLC-MS was supported by a decrease in inhibitory activity of protein phosphatase 1 (Figure 5). This indicates that the ozonolysis products are less toxic than parent MCs. The inhibitory effect decreased after 2 min of ozonolysis of MC-LR and MC-RR. For 1 L min^−1^ ozonolysis samples of MC-LR, the inhibitory activity reduced from 101% at 0 min to 23% at 2 min. Similarly, for 3 L min^−1^, it reduced from 96 to 21%. However, in case of 2 L min^−1^, the inhibitory activity decreased from 95 to 65% after 2 min, which was subsequently reduced to 8% after 4 min of treatment (Figure 5). After 4 min, the inhibitory activity was reduced in all the ozonolysis samples of MC-LR. All ozonolysis samples of MC-RR (at air flow rates of 1 L min^−1^, 2 L min^−1^ and 3 L min^−1^) showed a substantial decrease in the inhibitory activity of protein phosphatase 1 when sampled after just 2 min of ozonolysis treatment (Figure 5).

### Ozone treatment of *M. aeruginosa* cells

In addition to guidelines for the limit of MC-LR within drinking water (1 μg L^−1^), the WHO has also proposed two cyanobacteria alert levels for management of water resources, WHO Alert Level 1 is 2,000 cells mL^−1^ and WHO Alert Level 2 is 100,000 cells mL^−1^ (Chorus and Bartram, 1999). Therefore, removal of toxin containing cells would be a prerequisite to any toxin degradation technology. During water treatment, cyanobacterial cells can be removed via coagulation, and studies have shown that coagulants have little impact on cell integrity and therefore toxins are not released (Chow et al., 1999). However, the cyanobacteria can accumulate within sludge in sedimentation tanks or clarifiers, further risking cyanotoxin release as cells lyse. A combined treatment method, which can destroy cyanobacteria and release cyanotoxins prior to their degradation, would therefore provide considerable benefit. Chlorine treatment has been shown to damage the cell surface of *M. aeruginosa* cells and release intracellular toxins previously (Lam et al., 1995). Jurczak et al. applied chlorine dioxide treatment to *M. aeruginosa* and *M. wesenbergii* cells within reservoir water and showed an increase in extracellular MC as cells were degraded (Jurczak et al., 2005). However, the detrimental impact of using chemicals means alternative methods such as ozonation would be preferable, although it has been demonstrated that *Microcystis* is relatively more resistant to direct ozonation compared to three other genera of cyanobacteria tested, *Anabaena, Aphanizomenon*, and *Pseudanabaena* (Zamyadi et al., 2015). It was hypothesised that cell surface to volume ratio could be a contributing factor to this difference, though it could also be biochemical variations in the cell wall, and hence the composition of target molecules for the ozone.

**Figure 6:**
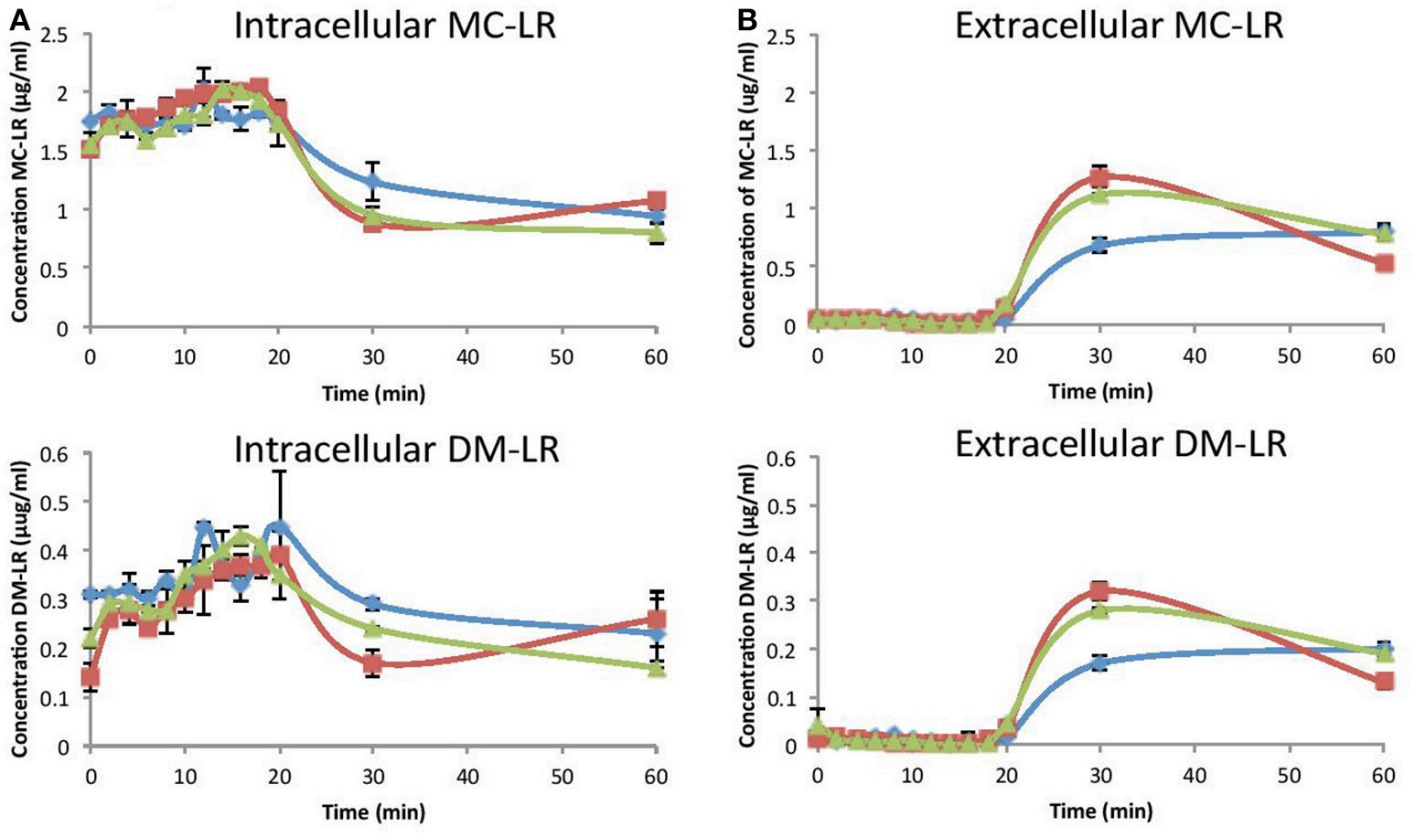
Intracellular and extracellular microcystin concentration at an air flow 1 L min^−1^ (0.199 ppm O_3_; Blue), 2 L min^−1^ (0.265 ppm O_3_; red) and 3 L min^−1^ (0.258 ppm O_3_; green) inside the cells **(A)** and in the media **(B)**. Top: MC-LR, Bottom: DM-LR (*n* = 3).

In order to evaluate the impact of ozonolysis treatment on *M. aeruginosa* cells using the plasma microreactor, intracellular and extracellular MC-LR and DM-LR levels were quantified in each flow rate experiment (Figure 6). *M*. *aeruginosa* was chosen as the model species here as they form the most prevalent toxic blooms globally. The initial concentration of intracellular MC-LR increased slightly during the first 18 min in all three flow rates, although this small change is attributed to potential cell clumping which was observed within the samples (data not shown). Clumping can be caused by oxidation-induced changes to the external cell structure, extracellular organic matter and degradation products of this (Zamyadi et al., 2013). Upon reaching MC-LR concentrations of 1.75-2 μg ml^−1^ in the defined culture volume, a rapid reduction in MC-LR concentrations were observed over the following 10 min, with faster reduction rate at flow rates of 2 and 3 L min^−1^ compared to 1 L min^−1^. Levels reached ~1 μg ml^−1^ after 60 min (Figure 6). This was further evidenced with a concordant increase in extracellular MC-LR after 20 min, where lysed cyanobacterial cells released MC-LR into the media leading to a large increase in MC-LR concentrations. Ozone flow rates of 2 and 3 L min^−1^ led to almost double the concentration of MC-LR in the media compared to 1 L min^−1^ ozone, implying higher levels of cell lysis. Surprisingly, the MC-LR and DM-LR concentrations at 60 min were higher than expected, and further investigation with more dynamic measurements is required to explain this.

Similar patterns of intracellular and extracellular changes in concentration levels of DM-LR were obtained although total concentrations were less. Intracellular levels varied at time point zero for the different flow rates, again indicating clumping. Intracellular levels of DM-LR reduced at similar rates with 1, 2 and 3 L min^−1^ flow rates from 20 to 30 min to between 0.15 and 0.3 μg ml^−1^. During this time, extracellular DM-LR increased at reciprocal levels, highest at 2 L min^−1^ ozone and lowest at 1 L min^−1^ (Figure 6).

During treatment of *M. aeruginosa* cells, images were taken at the lowest and highest ozone flow rates (1 and 3 L min^−1^ respectively) and these are shown in the Supplementary Materials (Figure S3). The appearance of blue pigment phycocyanin implies cell lysis, with 3 L min^−1^ ozone treatment leading to faster lysis compared to 1 L min^−1^ ozone treatment, confirming the intracellular and extracellular MC-LR and DM-LR concentration changes. In the Zamyadi et al. study (2013), over an order of magnitude lower cell densities were used (197,000 to 1,282,000 cells mL^−1^) compared to our study (12 million cells mL^−1^), but ozone concentrations were also a lot less (2–5 ppm vs. 20–27 ppm). They found that ~50% of *Microcystis* cells lysed after exposure to 2–5 mg L^−1^ ozone (2–5 ppm), with a contact time of 10 min. Using our plasma microreactor, cell lysis appeared to maximise between 20 and 30 min based on the sudden increase in extracellular MC's (Figure 6) and appearance of phycocyanin (Figure S3). It has been shown previously that toxin removal is strongly dependent on the concentration of ozone and Keijola et al. (1988) showed that ozonation at 1 mg L^−1^ was sufficient to remove all microcystins in the conditions they tested (Keijola et al., 1988).

## Conclusions

Although AOPs such as ozonation have emerged as effective methods to lyse cyanobacterial cells and degrade their associated cyanotoxins, current technologies suffer from high capital and operating costs. To overcome these drawbacks, a low temperature plasma DBD reactor was designed and constructed which incorporated the capability to deliver ozone through fluidic oscillator-generated microbubbles. This technology not only reduces operation costs but it was demonstrated here the smaller bubble size led to rapid degradation rates of exemplar cyanotoxins, MC-LR and MC-RR, at a flow rate at low as 1 L min^−1^. A survey of by-products also showed a reduction in concentrations, and importantly, reduced toxicity, evident through enzymatic assays. Ozonation of the MCs produced different degradation products reflecting structural variations. The lysis of *M. aeruginosa* cells after 20 min was apparent through increased detection of extracellular microcystins MC-LR and DM-LR, and increasing the flow rate indicated increased cell lysis. However, the oxidation rate is more difficult to determine from whole cells as it is highly dependent on the rate of toxin release from damaged cells as well as the presence of organic matter. This is the first study to combine these low cost technology advances for the treatment of toxic cyanobacterial species. However, more optimisation would need to be undertaken for application in natural waters with varied cyanobacterial compositions and concentrations, as well as other dissolved organic matters. Also, this work was undertaken in batch mode, whereas a continuous flow reactor would be required for scale up in the field.

## Author contributions

This study was devised by JP, LL, and CE. It was designed and coordinated by all authors. DK designed and constructed the plasma microreactor and WZ provided guidance on use of the fluidic oscillator. AS, CE, and LL undertook all the practical aspects of running the plasma microreactor and HPLC work. JP wrote the manuscript with editing from CE.

### Conflict of interest statement

The authors declare that the research was conducted in the absence of any commercial or financial relationships that could be construed as a potential conflict of interest.
