# Supplementary material for: Cell Lysis and Detoxification of Cyanotoxins Using a Novel Combination of Microbubble Generation and Plasma Microreactor Technology for Ozonation
Source: Front Microbiol. 2018 Apr 5;9:678. doi: 10.3389/fmicb.2018.00678 (PMC5895700; doi:10.3389/fmicb.2018.00678)
Supplement: Supplementary file 1 [file DataSheet1.DOCX]

Supplementary material

|  |  |
| --- | --- |

| Air flow rate (L min^-1^) | Ozone concentration (ppm) |
| --- | --- |
| 1.0 | 20 |
| 2.0 | 27 |
| 3.0 | 26 |

Figure S1: Ozone Concentrations measured at different flow rates of air.

|  |  |
| --- | --- |

| Air flow rate (L min^-1^) | Maximum bubble diameter (mm) |
| --- | --- |
| 1.0 | 0.56-0.60 |
| 2.0 | 0.66-0.70 |
| 3.0 | 0.76-0.80 |

Figure S2: Bubble size distribution measured at different flow rates of air.


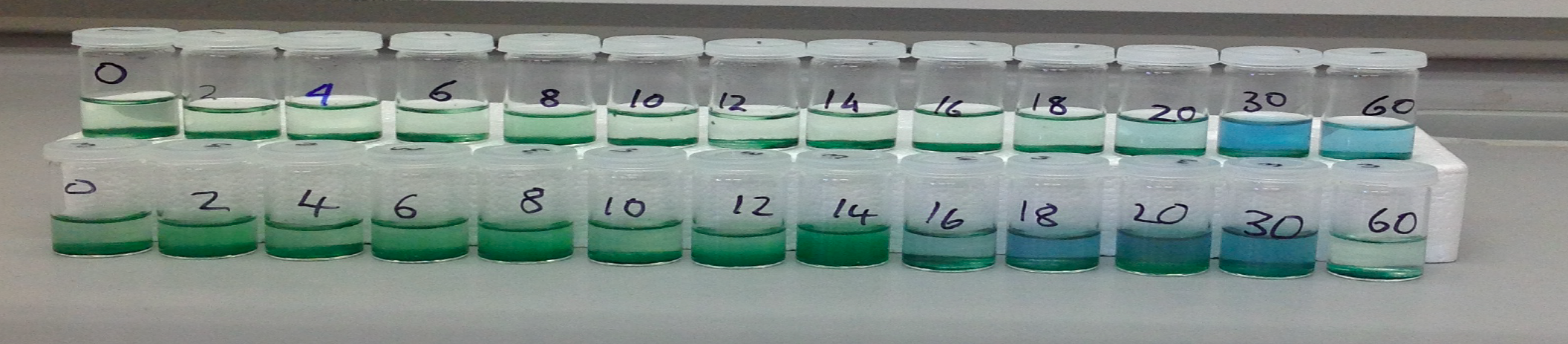


Figure S3: A visual representation of *M*. *aeruginosa* cells following ozonolysis treatment at two flow rates (top line is 1 L min^-1^ and bottom line is 3 L min^-1^). Sampling times are shown on the containers (mins).

Figure S4: Calibration Curve of Indigo Standard Solution
